# Nurses’ Perception of the Hospitals’ Spiritual Climate and Its Influence on Their Organizational Commitment: A Cross‐Sectional and Correlational Study

**DOI:** 10.1155/jonm/5163550

**Published:** 2026-03-31

**Authors:** Małgorzata Nagórska, Martin Červený, Magdalena Rękas, Aneta Lesiak, Zdzisława Chmiel, Mária Zamboriová, Silvia Danková, Tatiana Hrindová, Jonas Preposi Cruz

**Affiliations:** ^1^ Faculty of Health Sciences and Psychology, Collegium Medicum, University of Rzeszow, Rzeszów, Poland, ur.edu.pl; ^2^ Department of Nursing Care, Faculty of Medicine, Pavol Jozef Šafárik University in Košice, Košice, Slovak Republic, upjs.sk; ^3^ Institute of Social Sciences and Healthcare Blessed P. P. Gojdič in Prešov, St. Elizabeth University of Health and Social Work in Bratislava, Bratislava, Slovak Republic; ^4^ Department of Medicine, School of Medicine, Nazarbayev University, Astana, Kazakhstan, nu.edu.kz

## Abstract

**Background:**

The spiritual climate in the workplace has become more important in recent years, especially regarding organizational performance and employee well‐being. However, its effect on nurses’ organizational commitment in Poland and Slovakia has not yet been studied.

**Aim:**

This study aimed to examine the influence of hospital’s spiritual climate on organizational commitment as perceived by nurses in Slovakia and Poland.

**Design:**

Cross‐sectional and correlational study.

**Methods:**

The research involved a convenience sample of 390 nurses from Poland and Slovakia. The online questionnaire consisted of three parts: sociodemographic characteristics of respondents, Spiritual Climate Scale (SCS), and Three‐Component Model (TCM) Employee Commitment Survey. A standard multiple linear regression model was performed to investigate the influence of spiritual climate and sociodemographic variables on each of the TCM survey subscales.

**Results:**

Over half of the respondents (53.3%) experienced positive sensations according to the spiritual climate at their hospitals, but only one in three (33.1%) felt encouraged to express their spirituality at work. Affective commitment scored highest among nurses, followed by normative and continuance commitment. Regression models showed significant predictors, with managerial role, age, education, nationality, and perceived spiritual climate influencing commitment levels across subscales.

**Conclusion:**

The study showed that a positive hospital spiritual climate leads to higher organizational engagement, according to nurses from two European countries.

**Implications for Nursing Management:**

Hospital policymakers, administrators, and nursing leaders should consider integrating interventions and policies that promote a favorable spiritual climate in hospitals to ensure more committed nurses.

## 1. Introduction

The spiritual climate in the workplace has gained importance in recent years, particularly in the context of organizational performance and employee well‐being [1–4]. It is understood as the organizational readiness and favorable conditions that support spirituality [4]. This involves combining shared insights into spiritual values and their effective communication in the workplace [1].

The concept of a spiritual climate in the context of a hospital refers to the collective perception of healthcare workers regarding the spiritual atmosphere in their workplace. This includes the attitudes and interactions that nurses and other staff members experience when interacting with their colleagues and patients. A spiritual climate is characterized by an atmosphere of respect, openness, and support, in which individuals feel encouraged to express their views and ideas freely [3]. A spiritual climate is understood as an environment that not only respects individual opinions but also motivates individuals to fully dedicate themselves to their work [3].

This supportive atmosphere is crucial in healthcare settings, as it significantly impacts employee engagement, job satisfaction, and the overall quality of care provided to patients [1]. A positive spiritual climate enhances nurses′ engagement in their work, mental empowerment, and overall well‐being [4, 5].

A high level of spiritual climate correlates with greater emotional involvement and a sense of meaning at work, which is an important component of organizational commitment. Employees who feel that their spiritual needs are acknowledged and respected are more likely to express their willingness to stay in the organization and identify more closely with its mission and values [4].

In addition, an organization’s ability to cultivate a positive spiritual climate can greatly mediate staff stress and burnout levels, leading to improved patient care and reduced turnover rates among nurses [2, 3, 5]. By creating a caring spiritual environment, healthcare institutions can effectively mobilize and enhance the work experience of nurses, thereby improving the quality of care they provide to their patients [6, 7].

Spiritual climate, understood as an organizational atmosphere that supports spiritual values and a holistic approach to care, also plays an important role in shaping a sense of belonging and meaning of work among nursing staff [4]. A positive spiritual climate fosters the building of trust, respect, and emotional security, which are key components of organizational commitment [8]. Organizational commitment is the level of identification and the attitude of an employee toward the organization in which he or she is employed [9]. Organizational commitment is the individual’s mental connection to the organization [10]. There are three main components of this engagement. The first is affective commitment (AC), which refers to an emotional connection with the organization, identification with its goals, and active participation in its functioning. The second dimension is continuous commitment (CC), resulting from the belief in the costs or consequences of leaving the organization. The third aspect, i.e., normative commitment (NC), reflects an internal sense of obligation to remain in the organizational structures due to the norms and values recognized by the employee [10, 11].

Many studies confirmed that spirituality in the workplace influences organizational engagement [12–15]. For example, when nurses see their workplace as spiritually supportive, they are more likely to internalize the organization’s goals and values, which translate into stronger affective and normative engagement [16]. Therefore, developing the spiritual dimension of organizational culture can be a strategic tool in increasing employee loyalty and reducing staff turnover [3]. Also, institutions with a higher level of “spiritual saturation” are characterized by better cooperation in teams, greater trust in leaders, and a lower level of burnout, which indirectly promotes an increase in organizational commitment [3].

Hence, the spiritual climate of hospitals is a concept that plays a key role in shaping the job satisfaction of healthcare workers. By fostering a supportive and respectful workplace, a spiritual climate can lead to greater nurse engagement and a higher standard of patient care [2–4]. A spiritual climate is critical to supporting a workplace where people can thrive and thus ultimately improve treatment outcomes and patient satisfaction [3, 5]. Our study aims to investigate whether the organizational involvement of nurses in Poland and Slovakia is related to the spiritual climate of medical facilities.

### 1.1. Aim

This study aimed to investigate the influence of a hospital’s spiritual climate on organizational commitment as perceived by nurses in Slovakia and Poland.

## 2. Materials and Methods

### 2.1. Design

This study employed a quantitative design, specifically a cross‐sectional and correlational design, to examine the influence of the spiritual climate of hospitals on nurses’ organizational commitment.

### 2.2. Ethical Consideration

The study protocol was submitted and reviewed by the Research Ethics Committee of Pavol Jozef Šafárik University in Košice, Medical faculty (Reg. No: 12N/2025). The conduct of the study strictly adhered to the ethical principles for conducting research studies involving human subjects, as outlined in the Declaration of Helsinki and the policies and procedures of the Research Ethics Committee. During the recruitment process, potential participants were provided with sufficient information about the study to ensure informed decision‐making. They were informed about the voluntary nature of their participation. Participants also had the opportunity to ask questions or seek clarification from the researchers by contacting them through email. Those who consented signed an electronic informed consent. The privacy of respondents and the confidentiality of the data were protected throughout the study. The completed surveys were kept secure, and the data were analyzed and reported in aggregate.

### 2.3. Settings and Sample

The study surveyed nurses from Poland and Slovakia using a convenience sampling technique for sample selection. A total of 390 nurses participated in the study, which exceeds the required sample size of 123 nurses calculated with G^∗^Power 3.1 for a multiple regression analysis. This analysis involved 11 predictor variables and aimed to achieve a statistical power of at least 80% in detecting a hypothesized medium effect size (*f* ^2^ = 0.15) with a 0.05 margin of error.

The inclusion criteria were as follows: Participants were required to have at least 6 months of experience at their current public or private hospital, be employed either full‐time or part‐time, and have direct reporting relationships under leadership within the organization. Excluded from the study were nurses on temporary contracts or internships, as well as healthcare professionals outside of nursing roles.

### 2.4. Instrument

The researchers used an online survey to gather data for the study. The survey is divided into three main parts, each measuring the key variables of the study.

Part 1 included questions about the respondents’ sociodemographic information. This section collects data on age (in years), gender, marital status, area of residence, religion, highest educational qualification, current hospital position, total years of clinical nursing experience, years of experience at the present hospital, and area of practice.

Part 2 featured the Spiritual Climate Scale (SCS) developed by Doram et al. [3]. The SCS is a four‐item scale designed to evaluate the spiritual climate of clinical settings, as perceived by healthcare workers. The items include the following:

1. “I am encouraged to express spirituality in this clinical area.”
2. “My spiritual views are respected in this clinical area.”
3. “My spirituality has a comfortable home in this clinical area.”
4. “A diverse set of spiritual views are accepted in this clinical area.”

Each item was rated using a 5‐point Likert scale, where 1 indicates “*disagree strongly*” and 5 indicates “*agree strongly*.” To calculate the score, the mean of the four items was computed, then 1 was subtracted, and the result was multiplied by 25. This scoring formula yielded a potential score range from 0 to 100, with higher scores reflecting a more positive spiritual climate. Additionally, the “percent agreement” (or “percentage reporting good spiritual climate”) was calculated as the percentage of responses that fall into “*agree slightly*” and “*agree strongly*” categories for each item. A higher percentage indicated a “*good spiritual climate*.” The SCS exhibited good validity and reliability, demonstrated by Cronbach’s alpha of 0.863 [3]. This scale has been utilized in various studies among clinical nurses [2, 4, 17]. In this study’s sample, the computed Cronbach’s alpha was 0.84.

Part 3 consisted of the Three‐Component Model (TCM) Employee Commitment Survey developed by Allen and Meyer [18]. This scale measured three forms of employees’ organizational commitment: “desire‐based commitment” (AC), “obligation‐based commitment” (NC), and “cost‐based commitment” (continuance commitment). The original version of the tool contained 24 items with response options on a 7‐point Likert scale (1 = *strongly disagree*, 2 = *disagree*, 3 = *slightly disagree*, 4 = *undecided*, 5 = *slightly agree*, 6 = *agree*, and 7 = *strongly agree*). For this study, a shorter version consisting of 18 items with similar response options was used [19]. This shorter version included three subscales that assess the three types of organizational commitment, with each subscale containing six items. Four of the six items in each subscale were negatively worded; therefore, they were reverse‐scored before the final score computation. Subscale scores were calculated by averaging the scores of the six items, while the overall score was obtained by averaging the scores of all 18 items. Higher mean scores indicated higher organizational commitment. The scale’s developers advised against computing an overall mean score for the scale, recommending that each subscale be treated individually. The scale has demonstrated excellent validity and reliability [19]. In this study, Cronbach’s alpha values were 0.80 for the “Affective Commitment Subscale,” 0.81 for the “Continuance Commitment Subscale,” and 0.78 for the “Normative Commitment Subscale.” Overall Cronbach’s alpha for the entire scale was 0.87.

### 2.5. Translation and Pilot Testing of the Tool

The SCS and the TCM surveys were translated into Slovak and Polish using the forward–backward translation method [20]. This translation process followed five stages: Translation, Synthesis, Back translation, Expert review, and Pretesting. Two forward independent translations were performed from English to the Slovak and Polish languages. Then, the two independent forward translations were synthesized into a single Slovak and Polish version. The Slovak and Polish versions were back‐translated to English by two independent translators (for each version). The Slovak and Polish versions and the back‐translated versions were presented to a panel of experts with three members for review. The panel evaluated the content validity of the translated versions. Then, the final translated versions were pilot tested among 50 nurses for each version.

### 2.6. Data Collection

The survey was conducted from March to June 2025 as an online survey among professionally active nurses who met the study’s inclusion criteria. The questionnaire was made available through Microsoft Office Forms for nurses in Poland and Slovakia. Participants had to indicate their agreement or disagreement with the informed consent statement presented at the beginning of the survey to participate. The introduction provided information about the research’s purpose. Participants were also informed that their participation was voluntary; they were told that they could withdraw at any time without providing a reason or facing any consequences.

### 2.7. Statistical Analysis

SPSS Version 25.0 (IBMCorp., Armonk, NY, USA) was used for data analysis. We calculated descriptive statistics, including means, standard deviations, frequency, median interquartile range (IQR), and percentages, to analyze the sociodemographic variables, spiritual climate, and organizational commitment. We employed the Mann–Whitney *U* test, the Kruskal–Wallis *H* test, and the Spearman rank correlation to examine the relationships between sociodemographic characteristics and nurses’ perceptions of spiritual climate, as appropriate.

A standard multiple linear regression model was performed to investigate the influence of spiritual climate and sociodemographic variables on each of the TCM survey subscales. Predictor variables with nominal or categorical data were dummy‐coded before being entered into the regression analysis. The data met the assumptions required for conducting multiple linear regression analyses. Scatterplots of the residuals and predicted values indicated a linear relationship. Multicollinearity was not an issue, as evidenced by Tolerance values greater than 0.10 and Variance Inflation Factor (VIF) values less than 10. Histograms and Q‐Q plots were used to assess homoscedasticity, providing evidence that the residuals approximately followed a normal distribution. A *p* value of less than 0.05 was considered statistically significant.

## 3. Results

### 3.1. Characteristics of the Respondents

A total of 390 nurses completed the survey, with participants drawn from both countries. As shown in Table 1, 50.5% of the respondents were from Poland, while 49.5% were from Slovakia. The age of the participants ranged from 22 to 65 years, with a mean age of 39.54 years (SD = 9.78) and a median age of 40.00 years (IQR = 15.00). The majority of respondents were female (90.5%), married (57.9%), and lived in a city or town (55.9%). Most identified as Roman Catholic (81.8%) and worked as bedside nurses (79.0%). Nearly half of the respondents (45.9%) held a bachelor’s degree in nursing. The participants were employed in various practice areas, with the highest proportion working in the Surgical Department/Clinic (23.8%) and the lowest in the Oncology Department (1.3%). The mean years of experience as a nurse was 14.54 years (SD = 11.01), with a median of 12.00 years (IQR = 17.00). The mean years of experience in the current hospital was 10.91 years (SD = 9.48), with a median of 8.00 years (IQR = 12.50).

**TABLE 1:** Characteristics of the respondents (*n* = 390).

Variable	Minimum	Maximum	Mean	SD
Age	22.00	65.00	39.54	9.78
Years of experience as a nurse	1.00	45.00	14.54	11.01
Years of experience as a nurse in the present hospital	1.00	45.00	10.91	9.48

	***n***	**%**		

Country				
Poland	197	50.5		
Slovakia	193	49.5		
Gender				
Male	37	9.5		
Female	353	90.5		
Marital status				
Single	108	27.7		
Married	226	57.9		
Divorced/separated/widow/er	56	14.4		
Type of community				
City/town	218	55.9		
Village	172	44.1		
Religion				
Roman Catholic	319	81.8		
Greek Catholic	31	7.9		
Evangelical/Protestant	14	3.6		
Orthodox	6	1.5		
Jehovah’s Witness	5	1.3		
Atheist	15	3.8		
Education				
Secondary school of nursing/vocational nursing/nursing diploma	70	17.9		
Bachelor’s degree in nursing	179	45.9		
Masters	134	34.4		
Doctorate	7	1.8		
Role in the hospital				
Bedside nurse	308	79.0		
With managerial/leadership role	82	21.0		
Practice area				
Internal department/clinic	55	14.1		
Surgical department/clinic	93	23.8		
Geriatric ward/clinic	38	9.7		
Orthopedic department/clinic	34	8.7		
Intensive care and anesthesiology ward/clinic	51	13.1		
Neurological ward/clinic	37	9.5		
Gynecology and obstetrics department/clinic	19	4.9		
Pediatric department/clinic	19	4.9		
Psychiatric department/clinic	14	3.6		
Oncology	5	1.3		
Others	25	6.4		

Abbreviation: SD = standard deviation.

### 3.2. Hospital’s Spiritual Climate and Associated Factors

The overall mean score of the nurses in the SCS was 62.79, with a standard deviation of 21.82. More than half of the nurses reported a positive spiritual climate in their hospitals regarding how their spiritual beliefs are respected (53.3%), how their spirituality finds a comfortable home (50.3%), and the acceptance of diverse spiritual views (56.4%) in their clinical areas. However, only 33.1% of the nurses felt encouraged to express their spirituality in the workplace (see Table 2).

**TABLE 2:** Descriptive analysis results of the study variables (*n* = 390).

Variable	Range	Mean (SD)
Organizational commitment		
1. Affective commitment	1.50–7.00	4.64 (1.06)
2. Continuance commitment	1.00–7.00	4.23 (1.18)
3. Normative commitment	1.17–7.00	4.35 (1.12)
Overall spiritual climate score	0.00–100.00	62.79 (21.82)

	**Percentage reporting good spiritual climate**	
	***n***	**%**

1. I am encouraged to express spirituality in this clinical area.	129	33.1
2. My spiritual views are respected in this clinical area.	208	53.3
3. My spirituality has a comfortable home in this clinical area.	196	50.3
4. A diverse set of spiritual views are accepted in this clinical area.	220	56.4

Abbreviation: SD = standard deviation.

In Table 3, the bivariate analyses indicated that factors such as country of residence, gender, role in the hospital, and organizational commitment were associated with the nurses’ perceptions of their hospital’s spiritual climate. Nurses from Poland reported a more positive perception of the spiritual climate compared to those from Slovakia (*p* = 0.013). Male nurses had better perceptions than their female counterparts (*p* = 0.039). Bedside nurses perceived the spiritual climate more negatively than nurses in managerial or leadership roles (*p* = 0.015). Additionally, the nurses’ perceptions of the spiritual climate were positively associated with their affective (*p* < 0.001), continuance (*p* = 0.004), and normative (*p* < 0.001) organizational commitment.

**TABLE 3:** Factors associated with the nurses’ perceptions of their hospital’s spiritual climate (*n* = 390).

Variable	Mean rank	Statistical test	*p*
Age		*rho* = −0.03	0.574
Years of experience as a nurse		*rho* = −0.03	0.586
Years of experience as a nurse in the present hospital		*rho* = −0.08	0.115
Country			
Poland	209.46	*U* = 16261.00	0.013^∗^
Slovakia	181.25		
Gender			
Male	231.68	*U* = 5192.00	0.039^∗^
Female	191.71		
Marital status			
Single	209.93	*H* = 2.51	0.285
Married	189.41		
Divorced/separated/widow/er	192.24		
Type of community			
City/town	187.38	*U* = 16977.00	0.107
Village	205.80		
Religion (*n* = 379)			
Roman Catholic	190.13	*H* = 0.31	0.958
Greek Catholic	196.84		
Evangelical/Protestant	180.96		
Atheist	181.60		
Education			
Secondary school of nursing/vocational nursing/nursing diploma	191.54	*H* = 1.85	0.396
Bachelor’s degree in nursing	189.03		
Masters	205.68		
Doctorate			
Role in the hospital			
Bedside nurse	188.38	*U* = 10345.50	0.015^∗^
With managerial/leadership role	222.24		
Nurses’ organizational commitment			
Affective commitment		*rho* = 0.29	< 0.001^∗∗∗^
Continuance commitment		*rho* = 0.10	0.044^∗^
Normative commitment		*rho* = 0.27	< 0.001^∗∗∗^

*Note:* Mean rank = average rank; *ρ* = Spearman’s rank correlation coefficient; *U* = Mann–Whitney *U* test; *H* = Kruskal–Wallis *H* test; rho = Spearman’s rank correlation; *p* = level of statistical significance: ^∗^ *p* < 0.05; *p* < 0.01; ^∗∗∗^ *p* < 0.001.

### 3.3. Nurses’ Organizational Commitment and the Influence of Hospital’s Spiritual Climate and Other Factors

In Table 2, it is evident that AC received the highest mean score among the nurses (*M* = 4.64, *SD* = 1.06), followed by NC (*M* = 4.35, *SD* = 1.12) and continuance commitment (*M* = 4.23, *SD* = 1.18). The regression models for the three subscales of the TCM survey demonstrated significant results, explaining 18.9% of the variance (*R* ^2^ = 0.212, adjusted *R* ^2^ = 0.189, *F*_(11,  378)_ = 9.23, *p* < 0.001) in the mean scores for the Affective Commitment Subscale, 15.0% (*R* ^2^ = 0.174, adjusted *R* ^2^ = 0.150, *F*_(11,  378)_ = 7.23, *p* < 0.001) for the Continuance Commitment Subscale, and 16.3% (*R* ^2^ = 0.187, adjusted *R* ^2^ = 0.163, *F*_(11,  378)_ = 7.89, *p* < 0.001) for the Normative Commitment Subscale.

Table 4 highlights that both the nurse’s position in the hospital and the hospital’s spiritual climate significantly influence nurses’ AC. Specifically, nurses in managerial or leadership positions (*ß* = 0.14, *p* = 0.021, 95% CI = 0.02–0.25) and those with more positive perceptions of the hospital’s spiritual climate (*ß* = 0.01, *p* < 0.001, 95% CI = 0.01–0.02) showed higher levels of AC.

**TABLE 4:** Influence of hospital’s spiritual climate and other factors on nurses’ affective commitment (*n* = 390).

Predictor variables	*ß*	SE‐*b*	Beta	*t*	*p*	95% confidence intervals		Tolerance	VIF
Country	0.19	0.10	0.09	1.90	0.058	−0.01	0.39	0.92	1.08
Age	0.01	0.01	0.12	1.65	0.100	0.00	0.03	0.38	2.61
Gender	−0.01	0.17	0.00	−0.06	0.955	−0.34	0.32	0.94	1.06
Marital status (Reference group: Married)									
Single	−0.17	0.13	−0.07	−1.25	0.212	−0.43	0.10	0.64	1.56
Divorced/separated/widow/er	0.24	0.15	0.08	1.56	0.119	−0.06	0.53	0.83	1.20
Type of community	0.19	0.10	0.09	1.86	0.064	−0.01	0.39	0.91	1.10
Education (Reference group: Bachelor’s degree)									
Secondary school of nursing/vocational nursing/nursing diploma	0.13	0.15	0.05	0.89	0.372	−0.16	0.42	0.72	1.39
Graduate program	−0.13	0.11	−0.06	−1.19	0.237	−0.35	0.09	0.82	1.23
Position in the hospital	0.14	0.06	0.11	2.31	0.021^∗^	0.02	0.25	0.87	1.15
Years of experience in the present hospital	0.01	0.01	0.09	1.51	0.131	0.00	0.02	0.55	1.82
Hospital’s spiritual climate	0.01	0.00	0.27	5.79	< 0.001^∗∗∗^	0.01	0.02	0.96	1.04

*Note:* The depended variable was the mean score in the Affective Commitment Subscale. *ß* = unstandardized regression coefficient; SE‐*b* = standard error; Beta = standardized regression coefficient; *t* = statistic for the regression coefficient; *p* = level of statistical significance: ^∗^ *p* < 0.05; *p* < 0.01; ^∗∗∗^ *p* < 0.001; *R* ^2^ = 0.212, adjusted *R* ^2^ = 0.189.

Abbreviations: CI, confidence interval; VIF, variance inflation factor.

For continuance commitment, Slovakian nurses reported higher commitment levels than their Polish counterparts (*ß* = 0.43, *p* < 0.001, 95% CI = 0.20–0.65). Additionally, older age was linked to increased continuance commitment (*ß* = 0.03, *p* = 0.004, 95% CI = 0.01–0.04). Nurses with a secondary school of nursing, vocational nursing, or nursing diploma exhibited better continuance organizational commitment than those with a bachelor’s degree (*ß* = 0.40, *p* = 0.018, 95% CI = 0.07–0.74). Furthermore, positive perceptions of the hospital’s spiritual climate were associated with better continuance commitment (*ß* = 0.01, *p* < 0.001, 95% CI = 0.00–0.01) (see Table 5).

**TABLE 5:** Influence of hospital’s spiritual climate and other factors on nurses’ continuance commitment (*n* = 390).

Predictor variables	*ß*	SE‐*b*	Beta	*t*	*p*	95% confidence intervals		Tolerance	VIF
Country	0.43	0.11	0.18	3.75	< 0.001^∗∗∗^	0.20	0.65	0.92	1.08
Age	0.03	0.01	0.22	2.91	0.004^∗∗^	0.01	0.04	0.38	2.61
Gender	−0.09	0.19	−0.02	−0.49	0.626	−0.47	0.29	0.94	1.06
Marital status (Reference group: Married)									
Single	0.05	0.15	0.02	0.32	0.753	−0.25	0.35	0.64	1.56
Divorced/separated/widow/er	−0.09	0.17	−0.03	−0.54	0.587	−0.43	0.24	0.83	1.20
Type of community	0.19	0.12	0.08	1.67	0.096	−0.03	0.42	0.91	1.10
Education (Reference group: Bachelor’s degree)									
Secondary school of nursing/vocational nursing/nursing diploma	0.40	0.17	0.13	2.38	0.018^∗^	0.07	0.74	0.72	1.39
Graduate program	−0.04	0.13	−0.02	−0.34	0.735	−0.29	0.21	0.82	1.23
Position in the hospital	0.06	0.07	0.05	0.92	0.357	−0.07	0.20	0.87	1.15
Years of experience in the present hospital	0.00	0.01	0.03	0.50	0.617	−0.01	0.02	0.55	1.82
Hospital’s spiritual climate	0.01	0.00	0.17	3.56	< 0.001^∗∗∗^	0.00	0.01	0.96	1.04

*Note:* The depended variable was the mean score in the Continuance Commitment Subscale. *ß* = unstandardized regression coefficient; SE‐*b* = standard error; Beta = standardized regression coefficient; *t* = statistic for the regression coefficient; *p* = level of statistical significance: ^∗^ *p* < 0.05; *p* < 0.01; ^∗∗∗^ *p* < 0.001; *R* ^2^ = 0.174, adjusted *R* ^2^ = 0.150.

Abbreviations: CI, confidence interval; VIF, variance inflation factor.

As shown in Table 6, nurses from Slovakia (*ß* = 0.37, *p* = 0.001, 95% CI = 0.15–0.58) and nurses holding a managerial or leadership role (*ß* = 0.13, *p* = 0.044, 95% CI = 0.00–0.26) reported significantly higher levels of NC than their counterparts. Advanced age (*ß* = 0.02, *p* = 0.026, 95% CI = 0.00–0.04) and having a positive perception of the hospital’s spiritual climate (*ß* = 0.02, *p* < 0.001, 95% CI = 0.01–0.02) were positively associated with greater NC among the surveyed nurses.

**TABLE 6:** Influence of hospital’s spiritual climate and other factors on nurses’ normative commitment (*n* = 390).

Predictor variables	*ß*	SE‐*b*	Beta	*t*	*p*	95% confidence intervals		Tolerance	VIF
Country	0.37	0.11	0.16	3.40	0.001^∗∗^	0.15	0.58	0.92	1.08
Age	0.02	0.01	0.17	2.23	0.026^∗^	0.00	0.04	0.38	2.61
Gender	0.00	0.18	0.00	−0.03	0.979	−0.36	0.35	0.94	1.06
Marital status (Reference group: Married)									
Single	−0.02	0.14	−0.01	−0.15	0.883	−0.31	0.26	0.64	1.56
Divorced/separated/widow/er	−0.15	0.16	−0.05	−0.93	0.351	−0.47	0.17	0.83	1.20
Type of community	0.16	0.11	0.07	1.50	0.135	−0.05	0.38	0.91	1.10
Education (Reference group: Bachelor’s degree)									
Secondary school of nursing/vocational nursing/nursing diploma	0.16	0.16	0.05	0.98	0.326	−0.16	0.47	0.72	1.39
Graduate program	−0.04	0.12	−0.02	−0.30	0.765	−0.27	0.20	0.82	1.23
Position in the hospital	0.13	0.06	0.10	2.02	0.044^∗^	0.00	0.26	0.87	1.15
Years of experience in the present hospital	0.00	0.01	−0.01	−0.12	0.904	−0.02	0.01	0.55	1.82
Hospital’s spiritual climate	0.02	0.00	0.32	6.67	< 0.001^∗∗∗^	0.01	0.02	0.96	1.04

*Note:* The depended variable was the mean score in the Normative Commitment Subscale. *ß* = unstandardized regression coefficient; SE‐*b* = standard error; Beta = standardized regression coefficient; *t* = statistic for the regression coefficient; *p* = level of statistical significance: ^∗^ *p* < 0.05; *p* < 0.01; ^∗∗∗^ *p* < 0.001; *R* ^2^ *= *0.187, adjusted *R* ^2^ *= *0.163. Cronbach’s alpha. Spiritual Climate Scale = 0.84. Affective Commitment Subscale = 0.80. Continuance Commitment Subscale = 0.81. Normative Commitment Subscale = 0.78. Overall = 0.87.

Abbreviations: CI, confidence interval; VIF, variance inflation factor.

## 4. Discussion

This study examined how nurses’ perceptions of their hospital’s spiritual climate influence their organizational commitment. The results demonstrate a nuanced understanding of the spiritual climate in nursing workplaces, revealing a range of perceptions among nurses regarding spirituality and its integration into care practices. The reported overall mean score suggests a moderate perception of the spiritual climate, with slightly more than half of the nurses acknowledging the presence of a respectful environment for their beliefs and diverse spiritual views in the workplace. Overall, nurses reported a generally supportive spiritual climate in their clinical settings, characterized by respect for spiritual beliefs and acceptance of diverse spiritual views. This finding aligns with previous research highlighting the critical role of workplace spirituality in fostering a nurturing environment for healthcare providers, which in turn may enhance their competence in delivering spiritual care [17, 21]. However, it is notable that only 33.1% of the nurses expressed feeling encouraged to articulate their spirituality at work, pointing to a potential gap between the perception of a supportive environment and the actual practices that enable spiritual expression. Various studies underscore the importance of creating spaces within healthcare settings that encourage nurses to integrate their spirituality into patient care without feeling restricted [22, 23]. This hesitance may stem from systemic barriers, such as organizational culture and the perceived prioritization of medical care over spiritual care interventions, as indicated by research suggesting a disconnect between nurses’ personal beliefs and their professional responsibilities [24].

The bivariate analyses shed light on several demographic and role‐related factors influencing nurses’ perceptions of the spiritual climate in their workplaces. Notably, nurses in Poland reported a more positive perception than those in Slovakia, which could reflect broader cultural attitudes toward spirituality in healthcare within different countries [25]. Gender differences were also significant, with male nurses indicating better perceptions than their female colleagues (*p* = 0.039). This disparity could illuminate underlying sociocultural dynamics that influence nurses’ comfort in expressing spirituality [26], as well as the need for tailored approaches in spirituality education that cater to different demographics [27]. Furthermore, the analysis indicates that the role of nurses significantly affects their perceptions of the spiritual climate, with bedside nurses expressing less positive views about it compared to those in leadership positions. This could be attributed to the additional responsibilities of leadership roles that may allow for more autonomy in integrating and practicing their spirituality into their practice [28].

The multiple linear regressions analyzed some demographic factors influencing nurses’ organizational commitment in the two countries. Being a nurse manager, older age, having a lower educational qualification, and being a nurse from Slovakia were associated with greater organizational commitment, at least in one dimension. Similar findings were reported by nurses from the Philippines, where nurse managers and older nurses showed higher organizational commitment. Conversely, nurses with higher educational qualifications (i.e., Master’s level) reported better organizational commitment [29]. A study from China also found similar results regarding position and age [30]. These studies suggest that nurses in leadership positions have better compensation and benefits, greater autonomy, more decision‐making involvement, and better career advancement opportunities than those who do not hold such positions—factors that literature indicates can improve organizational commitment [26, 30, 31]. These factors may also explain the findings in the current study sample. A previous study reported that younger Polish nurses experience more physical problems related to poor working conditions, which could negatively impact organizational commitment. The same study highlighted that older nurses benefit more from having a “voice at work” than younger nurses, a factor that enhances organizational commitment [32]. Job satisfaction was also lower among Slovak nurses without leadership roles, which is a predictor of organizational commitment and supports our current findings [33]. The differences in organizational commitment between countries may be complex to explain but could relate to common factors influencing commitment such as working conditions, benefits, potential career advancement, available support, leadership, and the social impact of the profession in each country. These factors, however, need further investigation in each country in future studies.

The positive association between nurses’ perceptions of the spiritual climate and their organizational commitment (affective *p* < 0.001; continuance *p* = 0.004; normative *p* < 0.001) supports the literature asserting that higher levels of workplace spirituality contribute to increased emotional investment in their organizations [28]. Nurses who recognize the alignment of their work environment with their spiritual values are likely to experience greater job satisfaction and reduced burnout, a finding that advocates for the intentional fostering of a spiritually supportive climate within healthcare institutions [30].

In summation, the findings reveal a complex interplay between personal spirituality, workplace environment, and organizational dynamics that shapes nurses’ perceptions and practices concerning spiritual care. Addressing the evident barriers to spiritual expression among nursing staff is crucial, as it may enhance overall patient care quality and the nurses’ job satisfaction.

### 4.1. Limitations of the Study

The study has several limitations that should be acknowledged when interpreting and applying its findings. The study’s design does not allow for examining the cause‐and‐effect relationship between variables. It only looked at the association between organizational spiritual climate and organizational commitment among nurses. Additionally, the study used convenience sampling, which limits how well the results can be applied to other nurses and hospitals in the two countries. Regarding the instrument, while we followed acceptable methods of linguistic adaptation for the tools used, a full psychometric analysis of the translated version was not conducted due to limited resources. Therefore, future research should evaluate the full validity and reliability of the translated tool. The initial reliability evidence reported in this study should be supported by a more comprehensive analysis of the tool’s psychometric properties. The self‐report nature of the study may have introduced some social desirability bias. The study focused solely on two European countries, so the results may not be representative of nurses from other nations. The examined factors related to spiritual climate and organizational commitment were limited to demographic and some work‐related variables. Future studies should consider exploring other factors that might influence these variables. Finally, the factors affecting the hospital’s spiritual climate and nurses’ organizational commitment were not examined by the country, which should be addressed in future research.

## 5. Conclusions

The study provided evidence on the influence of a positive hospital’s spiritual climate in ensuring stronger organizational commitment, as reported by nurses in two European countries. The study concludes that a hospital’s climate that encourages the expression of, respects, provides a comfortable home for, and accepts diverse spirituality may lead to better affective, continuance, and NC among nurses working in those hospitals. These findings underscore the critical role of workplace spirituality in nurses’ work outcomes, such as organizational commitment. Hospital policymakers, administrators, and nursing leaders should consider integrating interventions and policies that promote a positive spiritual climate in hospitals to ensure more committed nurses. We also conclude that several characteristics of nurses play important roles in ensuring their commitment to the organization, such as the nurses’ position in the hospital, age, educational qualification, and country of residence. These factors should be taken into account when understanding nurses’ organizational commitment and when developing interventions and policies to increase nurses’ level of commitment.

## Author Contributions

Conception or design of the work: Jonas Preposi Cruz, Martin Červený, and Małgorzata Nagórska.

Data collection: Małgorzata Nagórska, Martin Červený, Magdalena Rękas, Silvia Danková, Tatiana Hrindová, Aneta Lesiak, and Zdzisława Chmiel, Mária Zamboriová.

Analysis or interpretation of data: Jonas Preposi Cruz, Martin Červený, and Małgorzata Nagórska.

Drafting the work: Jonas Preposi Cruz, Martin Červený, and Małgorzata Nagórska.

Reviewing it critically for important intellectual content: Jonas Preposi Cruz, Martin Červený, Małgorzata Nagórska, and Mária Zamboriová.

## Funding

The study did not receive any form of funding.

## Disclosure

All authors gave final approval of the version to be published and agreed to be accountable for all aspects of the work.

## Conflicts of Interest

Jonas Preposi Cruz and Martin Červený served as the Guest Editors of the journal’s special issue titled “The Nexus of Workplace Culture, Spirituality, and Religiosity in Fostering Positive Clinical Work Environments for Nurses: Implications for Nursing Management.” However, they did not have any participation in the editorial handling of the manuscript. The other authors declare no conflicts of interest.

## Data Availability

The data that support the findings of this study are available from the corresponding author upon reasonable request.

## References

[1] Khari C. and Sinha S. , Impact of Workplace Spirituality on Knowledge Sharing Intention: A Conceptual Framework, Journal of Human Values. (2017) 23, no. 1, 27–39, 10.1177/0971685816673484, 2-s2.0-85011372851.

[2] Cruz J. P. , Alquwez N. , Albaqawi H. M. , Alharbi S. M. , and Moreno-Lacalle R. C. , Nurses’ Perceived Spiritual Climate of a Hospital in Saudi Arabia, International Nursing Review. (2018) 65, no. 4, 559–566, 10.1111/inr.12481, 2-s2.0-85053710347. 30239998

[3] Doram K. , Chadwick W. , Bokovoy J. , Profit J. , Sexton J. D. , and Sexton J. B. , Got Spirit? The Spiritual Climate Scale, Psychometric Properties, Benchmarking Data and Future Directions, BMC Health Services Research. (2017) 17, no. 1, 10.1186/s12913-017-2050-5, 2-s2.0-85013058515. PMC5303307 28189142

[4] Cruz J. P. , Alquwez N. , Mesde J. H. , Almoghairi A. M. A. , Altukhays A. I. , and Colet P. C. , Spiritual Climate in Hospitals Influences Nurses’ Professional Quality of Life, Journal of Nursing Management. (2020) 28, no. 7, 1589–1597, 10.1111/jonm.13113. 32743944

[5] Zhang Y. , Wu X. , Wan X. et al., Relationship Between Burnout and Intention to Leave Amongst Clinical Nurses: The Role of Spiritual Climate, Journal of Nursing Management. (2019) 27, no. 6, 1285–1293, 10.1111/jonm.12810, 2-s2.0-85069635649. 31144776

[6] Pio R. J. and Tampi J. R. E. , The Influence of Spiritual Leadership on Quality of Work Life, Job Satisfaction and Organizational Citizenship Behavior, International Journal of Law and Management. (2018) 60, no. 2, 757–767, 10.1108/IJLMA-03-2017-0028, 2-s2.0-85047663208.

[7] Fradelos E. , Alexandropoulou C. A. , Kontopoulou L. , Papathanasiou I. V. , and Tzavella F. , Factors. Affecting Greek Nurses’ Caring Behaviors: The Role of Nurses’ Spirituality and the Spiritual Climate of Hospitals, Journal of Religion and Health. (June 2022) 61, no. 3, 1816–1830, 10.1007/s10943-022-01503-x. 35044588

[8] Ivziku D. , Biagioli V. , Caruso R. et al., Trust in the Leader, Organizational Commitment, and Nurses’ Intention to Leave—Insights From a Nationwide Study Using Structural Equation Modeling, Nursing Reports. (2024) 14, no. 2, 1452–1467, 10.3390/nursrep14020109. 38921719 PMC11206282

[9] Callado A. , Teixeira G. , and Lucas P. , Turnover Intention and Organizational Commitment of Primary Healthcare Nurses, Healthcare. (2023) 11, no. 4, 10.3390/healthcare11040521. PMC9957010 36833055

[10] Dominic E. and Salim M. H. , A Study on the Role of Organizational Commitment and Perception Towards Organizational Justice and Fairness in Triggering Organizational Citizenship Behavior Among B School Faculty Members in Kerala, Rajagiri Management Journal. (2018) 12, 21–40.

[11] Grego-Planer D. , The Relationship Between Organizational Commitment and Organizational Citizenship Behaviors in the Public and Private Sectors, Sustainability. (2019) 11, no. 22, 10.3390/su11226395.

[12] Milliman J. , Czaplewski A. J. , and Ferguson J. , Workplace Spirituality and Employee Work Attitudes: An Exploratory Empirical Assessment, Journal of Organizational Change Management. (2003) 16, no. 4, 426–447, 10.1108/09534810310484172, 2-s2.0-0041410270.

[13] De Carlo A. , Dal Corso L. , Carluccio F. , Colledani D. , and Falco A. , Positive Supervisor Behaviors and Employee Performance: The Serial Mediation of Workplace Spirituality and Work Engagement, Frontiers in Psychology. (2020) 11, 10.3389/fpsyg.2020.01834. PMC7393218 32793085

[14] Ghamshadzahi P. and Nastiezaie N. , The Relationship Between Workplace Spirituality and Work Engagement With the Mediating Role of Organizational Loyalty (Case Study: Elementary School Teachers of Khash City), Knowledge & Research in Applied Psychology. (2020) 20, no. 78, 46–56.

[15] Chouhan V. S. , Examining Relationship of Workplace Spirituality with Work Engagement, Organizational Commitment and Trust, Vikalpa. (2025) 0, 10.1177/02560909251325386.

[16] Rodríguez-Fernández M. , Herrera J. , de Las Heras-Rosas C. , and Ciruela-Lorenzo A. M. , Practical Implications of the Organizational Commitment Model in Healthcare: The Case of Nurses, Journal of Nursing Management. (May 2024) 2024, 10.1155/2024/6455398. PMC11918622 40224884

[17] Albaqawi H. , Alquwez N. , Almazan J. et al., Workplace Spiritual Climate and Its Influence on Nurses’Provision of Spiritual Care in Multicultural Hospitals, Religions. (2019) 10, no. 2, 10.3390/rel10020118, 2-s2.0-85062830574.

[18] Allen N. J. and Meyer J. P. , The Measurement and Antecedents of Affective, Continuance and Normative Commitment to the Organization, Journal of Occupational Psychology. (1990) 63, 1–18, 10.1111/j.2044-8325.1990.tb00506.x, 2-s2.0-84986665106.

[19] Meyer J. P. , Allen N. J. , and Smith C. A. , Commitment to Organizations and Occupations: Extension and Test of a Three-Component Conceptualization, Journal of Applied Psychology. (1993) 78, no. 4, 538–555, 10.1037/0021-9010.78.4.538, 2-s2.0-23144467997.

[20] Beaton D. E. , Bombardier C. , Guillemin F. , and Ferraz M. B. , Guidelines for the Process of Cross-Cultural Adaptation of Self-Report Measures, Spine. (2000) 25, no. 24, 3186–3191, 10.1097/00007632-200012150-00014, 2-s2.0-0034671451. 11124735

[21] Alshehry A. , Spirituality and Spiritual Care Competence Among Expatriate Nurses Working in Saudi Arabia, Religions. (2018) 9, no. 12, 10.3390/rel9120384, 2-s2.0-85057241394.

[22] Hu Y. , Jiao M. , and Li F. , Effectiveness of Spiritual Care Training to Enhance Spiritual Health and Spiritual Care Competency Among Oncology Nurses, BMC Palliative Care. (2019) 18, no. 1, 10.1186/s12904-019-0489-3. PMC6880564 31771570

[23] Shaha M. , Ferrell B. , and Economou D. , Nurses’ Response to Spiritual Needs of Cancer Patients, European Journal of Oncology Nursing. (2020) 48, 10.1016/j.ejon.2020.101792. 32947158

[24] Ronaldson S. , Hayes L. , Aggar C. , Green J. , and Carey M. , Spirituality and Spiritual Caring: Nurses’ Perspectives and Practice in Palliative and Acute Care Environments, Journal of Clinical Nursing. (2012) 21, no. 15-16, 2126–2135, 10.1111/j.1365-2702.2012.04180.x, 2-s2.0-84864003901. 22788554

[25] Bolivar G. R. , Villaroman J. M. , Alshehri M. A. , and Balaria C. J. R. , Making Room for Faith: Art of Spiritual Care in an Islamic Country, IJAMRS. (2024) 4, no. 1, 1150–1155, 10.62225/2583049x.2024.4.1.2356.

[26] Labrague L. , McEnroe–Petitte D. , Achaso R. , Cachero G. , and Mohammad M. , Filipino Nurses’ Spirituality and Provision of Spiritual Nursing Care, Clinical Nursing Research. (2016) 25, no. 6, 607–625, 10.1177/1054773815590966, 2-s2.0-84994159089. 26084286

[27] Adib‐Hajbaghery M. , Zehtabchi S. , and Azizi-Fini I. , Iranian Nurses’ Professional Competence in Spiritual Care in 2014, Nursing Ethics. (2015) 24, no. 4, 462–473, 10.1177/0969733015600910, 2-s2.0-85020508753. 26311490

[28] Kazemipour F. , Amin S. , and Pourseidi B. , Relationship Between Workplace Spirituality and Organizational Citizenship Behavior Among Nurses Through Mediation of Affective Organizational Commitment, Journal of Nursing Scholarship. (2012) 44, no. 3, 302–310, 10.1111/j.1547-5069.2012.01456.x, 2-s2.0-84865655857. 22804973

[29] Labrague L. J. , McEnroe–Petitte D. M. , Tsaras K. , Cruz J. P. , Colet P. C. , and Gloe D. S. , Organizational Commitment and Turnover Intention Among Rural Nurses in the Philippines: Implications for Nursing Management, International journal of nursing sciences. (2018) 5, no. 4, 403–408, 10.1016/j.ijnss.2018.09.001, 2-s2.0-85056395428. 31406855 PMC6626268

[30] Tang P. , Zhang X. , Feng F. et al., The Relationship Between Organizational Commitment and Work Engagement Among Clinical Nurses in China: A Cross‐Sectional Study, Journal of Nursing Management. (2022) 30, no. 8, 4354–4363, 10.1111/jonm.13847. 36196679

[31] Sikorska-Simmons E. , Predictors of Organizational Commitment Among Staff in Assisted Living, Gerontologist. (2005) 45, no. 2, 196–205, 10.1093/geront/45.2.196, 2-s2.0-15944380027. 15799984

[32] Van der Heijden B. I. , Houkes I. , Van den Broeck A. , and Czabanowska K. , I Just Can’t Take It Anymore: How Specific Work Characteristics Impact Younger Versus Older Nurses’ Health, Satisfaction, and Commitment, Frontiers in Psychology. (2020) 11, 10.3389/fpsyg.2020.00762. PMC7267024 32536884

[33] Kožuchová M. and Vargová A. , Selected Factors of Slovak Nurses Job Satisfaction, Central European Journal of Nursing and Midwifery. (2015) 6, no. 2, 260–266, 10.15452/CEJNM.2015.06.0013, 2-s2.0-84930587283.

